# Evidence for motivational interviewing in educational settings among medical schools: a scoping review

**DOI:** 10.1186/s12909-024-05845-w

**Published:** 2024-08-08

**Authors:** Leonard Yik Chuan Lei, Keng Sheng Chew, Chee Shee Chai, Yoke Yong Chen

**Affiliations:** Faculty of Medicine and Health Sciences, Universiti Malaysia Sarawak (UNIMAS), Kota Samarahan, Sarawak 94300 Malaysia

**Keywords:** Scoping review, Motivational interviewing, Motivational behaviour, Motivational change, Motivational enhancement, Medical education, Medical teaching

## Abstract

**Background:**

Motivational interviewing (MI) is a person-centred approach focused on empowering and motivating individuals for behavioural change. Medical students can utilize MI in patient education to engage with patients’ chronic health ailments and maladaptive behaviours. A current scoping review was conducted to 1) determine the types of MI (conventional, adapted, brief and group MI) education programs in medical schools, delivery modalities and teaching methods used; 2) classify educational outcomes on the basis of Kirkpatrick’s hierarchy; and 3) determine the key elements of MI education via the FRAMES (feedback, responsibility, advice, menu of options, empathy, self-efficacy) model.

**Methods:**

This scoping review was conducted via the framework outlined by Arksey and O’Malley. Two online databases, CINAHL and MEDLINE Complete, were searched to identify MI interventions in medical education. Further articles were selected from bibliography lists and the Google Scholar search engine.

**Results:**

From an initial yield of 2019 articles, 19 articles were included. First, there appears to be a bimodal distribution of most articles published between the two time periods of 2004--2008 and 2019--2023. Second, all the studies included in this review did not use conventional MI but instead utilized a variety of MI adaptation techniques. Third, most studies used face-to-face training in MI, whereas only one study used online delivery. Fourth, most studies have used a variety of interactive experiences to teach MI. Next, all studies reported outcomes at Kirkpatrick’s Level 2, but only 4 studies reported outcomes at Kirkpatrick’s Level 3. According to the FRAMES model, all studies (*n*=19; 100%) reported the elements of responsibility and advice. The element that was reported the least was self-efficacy (*n* = 12; 63.1%).

**Conclusion:**

Our findings suggest that motivational interviewing can be taught effectively in medical schools via adaptations to MI and a variety of teaching approaches. However, there is a need for further research investigating standardized MI training across medical schools, the adequate dose for training in MI and the implementation of reflective practices. Future studies may benefit from exploring and better understanding the relationship between MI and self-efficacy in their MI interventions.

## Background

Motivational interviewing (MI) is a person-centred approach that focuses on empowering and motivating individuals for behavioural change [1]. Undoubtedly, the empathetic approach of MI in clinical settings fosters a supportive environment that encourages discussion of the benefits of enhanced self-care [2]. In this context, MI practitioners utilize a set of essential skills encapsulated by the acronym “OARS”, which stands for O = open-ended questions, A = affirmations, R = reflections and S = summaries to promote active listening [3]. MI was developed primarily for the treatment of addiction disorders but has since progressed to include other physical and mental ailments as well [4]. In a study on MI interventions in alcoholism, Miller & Sanchez [68] identified six common motivational elements that should be covered, represented by the acronym “FRAMES”, where F = feedback (e.g., personalized feedback on the impacts of alcoholism on the client’s own experiences, as opposed to providing generic information); R = responsibility (e.g., empowering clients to make their own choices and take responsibility for their change process); A = advice (e.g., effectively given in a nondirective and noncoercive manner); M = menu (e.g., offering a variety of choices on transition methods and plans); E = empathy (e.g., rendering empathic, reassuring and reflective listening); and S = self-efficacy (e.g., supporting clients to succeed in a specified goal). This review used the FRAMES model to determine the key elements of MI education. FRAMES was a predecessor to MI and was initially designed to address drinking problems [5]; however, it is also used in other health issues, such as decreasing stroke risk [6], substance use screening and brief intervention [7]. The FRAMES model offers a structure that can be used to improve the delivery of MI by ensuring that key elements of MI are present in educational interventions.

### Mechanisms of motivational interviewing

Frey et al. [8] developed mechanisms of the motivational interviewing (MMI) framework and described the mechanisms of fidelity of practice in MI, including a technical component, a relational component and MI-inconsistent practices [8]. The technical component consists of the interviewer’s ability to evaluate the participant’s language relating to a specific behaviour change target and then build a conversation that evokes change talk. The relational component includes respect for the participant’s self-determination, appropriate empathy, and equal partnership. Non-MI consistent behaviours include confrontation, offering unsolicited advice, and persuasion. Additionally, it is important to identify and understand the mechanisms of change so that MI users and researchers can focus on these mechanisms during training, which can lead to improved outcomes and fidelity [8].

### Types of motivational interviewing

MI can be categorized into four types: conventional, adaptive, brief, and group. Conventional MI is an evidence-based approach and directive form of interviewing developed by Miller & Rollnick [9]. Throughout the course of MI, four important tasks occur: engaging (building mutual relationships), focusing (setting goals), evoking (developing clients’ motivations for change) and planning (negotiating change) [9]. In this review, the term conventional MI is defined as an approach that utilizes MI-consistent tasks and behaviours in multiple sessions that target an identified population of clients.

Adapted MI consists of culturally sensitive MI and digitally supported interventions that can be used as adjunct interventions to the primary behavioural program [10]. This review defines the term adapted MI to include any adaptations made to adapt MI culturally to the setting or delivered by technology through various types of technologies and content (e.g., computers, smartphones, applications, videos and audio). Additionally, it also includes adaptations made to structured curricula, such as using role plays or real patient interactions to facilitate the learning of MI.

Brief MI is a type of MI with varying lengths, ranging from 5--90 minutes in duration, emphasizing the lack of an accepted definition of brief MI [10]. This review defines the term brief MI as an MI that provides brief consultations centred on typically fewer sessions (e.g., 1--2 sessions) than conventional MI (e.g., 3--4 sessions or more).

Group MI can be defined as groups of clients that apply the MI spirit, processes and methods to increase motivation for change and promote beneficial collaboration among participants and practitioners in a shared location to encourage change [11]. This review defines the term group MI as MI that is adapted for group format and is MI consistent (e.g., applying MI principles, spirit and techniques in its delivery).

Additionally, MI can be used in patient education to help patients better handle their chronic health conditions and maladaptive behaviours. Therefore, behavioural change is vital in the recovery course of different mental and physical disorders, as a change to a healthier lifestyle has been shown to result in a significant decrease in chronic disease risk [12]. More than 120 studies have demonstrated the efficacy of MI in addressing a wide range of problematic behaviours, such as substance abuse and risky behaviour, as well as promoting healthy behaviours [13]. There is specific evidence regarding the effectiveness of MI across different health behaviours (substance abuse, risky behaviours and promoting health behaviours), according to the types of MI: conventional, adaptive, brief and group. For conventional MI, research has shown effectiveness in treating substance abuse [14], reducing risky behaviours in human immunodeficiency virus (HIV)-positive men [15] and promoting physical activity in older adults [16]. Adaptive MI has demonstrated its effectiveness in reducing alcohol problems in women [17], reducing risky sexual behaviours and psychological symptoms in HIV-positive older adults [18] and promoting self-management to reduce BMI and improve lifestyle adherence with a computer assistant [19]. Brief MI has been effective in reduction in alcohol misuse in college students with attention deficit hyperactivity disorder (ADHD) [20] and improvement in the engagement of physical activity in patients with low physical activity levels [21]. Research has revealed that group MI is effective in treating drug use among women [22], reducing risky sexual behaviour among adolescents [23] and improving self-efficacy and oral health behaviours among pregnant women [24].

Unhealthy lifestyle-linked behaviours characterize common preventable risk factors that lead to the majority of noncommunicable diseases and their associated mortality and morbidity [25]. MI provides an approach for healthcare providers to assist patients in investigating and resolving their ambivalence toward changing unhealthy lifestyle behaviour [27]. Studies have reported the effectiveness of teaching MI to medical students [4, 26, 28–30]. Therefore, considering the prevalence and widespread application of MI in health care settings, this underscores the importance of MI being taught in the initial stages of medical education.

In a recent systematic review, Kaltman and Tankersley [31] reviewed MI curricula in undergraduate medical education (UME) and revealed important findings. Their research findings suggest that generally being involved in an MI curriculum can be linked to enhanced MI-related knowledge and skills in the short term. Additionally, they noted that 1) the MI curricula were heterogeneous in nature; 2) the curricula were different in terms of timing, duration and number of sessions; 3) the curricula employed in studies were multiple pedagogies; and 4) the quality of the evaluations and research evidence varied. However, this review by Kaltman and Tankersley [31] was limited to reporting only on MI-specific outcomes such as knowledge, skills, attitudes towards, and self-efficacy in implementing MI. Kaltman and Tankersley [31] systematic review did not stratify and explore in detail studies on the types of MI (conventional, adaptive, brief, or group). Furthermore, the systematic review did not investigate the key elements of MI education as described by the FRAMES model. The scoping review aimed to bridge the knowledge gap on types of MI (conventional, adapted, brief, group MI) and key elements of MI education covered via the FRAMES model. Specifically, the objectives of this study were to 1) determine the types of MI education programs in medical schools, the delivery modalities, and the teaching methods used; 2) classify educational outcomes on the basis of Kirkpatrick’s hierarchy [32]; and 3) determine the key elements of MI education covered via the FRAMES model.

## Methods

### Procedure

This study adopted the methodological 5-step framework of Arksey and O’Malley for this scoping review. The five steps are as follows: 1) define our research objectives; 2) identify relevant studies; 3) identify studies based on our selection criteria; 4) chart and analyse the data; and 5) collate, summarize, and disseminate the results.

### Eligibility criteria

Relevant peer-reviewed articles on MI studies conducted in medical education settings, published in academic journals only, in the English language, with no time limit imposed on the publication period, were identified. Studies involving nonmedical students as well as grey literature, such as conference proceedings, technical reports, videos, and informal communications, were excluded. Studies in languages other than English were also excluded. The search strategy was guided by the methodology of Aromataris and Riitano [33]. The Boolean operators and keywords used in this search strategy were ("medical education" OR "medical teaching*" OR "medical graduate*" OR "medical postgraduate*” OR “medical student*”) AND ("motivational interview*" OR "motivational enhanc*" OR "motivational chang*" OR "motivational behavior”) AND ("psycholog*" OR "health*"). The search utilized databases from the Medical Literature Analysis and Retrieval System Online (MEDLINE Complete) and Cumulative Index of Nursing and Allied Health Literature (CINAHL Complete) databases via the EBSCOHost database search query, covering all study designs (i.e., quantitative, qualitative, and mixed studies). The protocol was developed a priori before the search process was conducted, including establishing the objectives and eligibility criteria for determining the studies selected. The reference lists of the selected studies were further checked for additional sources, including traditional and systematic reviews. Articles that met the eligibility criteria were selected through a consensus among the authors and were charted according to the Preferred Reporting Items for Systematic reviews and Meta-analysis extension for Scoping Reviews (PRISMA-ScR) guidelines [34]. The first author conducted the searches and screened the articles using the search strategy and the inclusion and exclusion criteria stated above. This process resulted in the identification of 59 articles. The decision process resulted in 19 studies for inclusion in this review based on the inclusion and exclusion criteria. The data were extracted and charted by the first author. Notably, the following data were extracted: 1) the study characteristics of the identified articles (publication year, country of origin, type of MI, and medical student phase) and 2) a detailed description of the key findings of the articles (i.e., author, year, objectives, participants, delivery, duration, teaching methods, assessments, and educational outcomes based on Kirkpatrick’s hierarchy). Proforma was developed by all the authors and used to extract and chart the data. The study characteristics are then charted in Table 1, and detailed descriptions of the key findings of the articles are charted in Table 2. The other authors assisted in identifying specific data elements to be charted onto Tables 1 and 2. All the authors contributed to analysing the charted data to ensure the consistency and accuracy of the analysis. The outcomes of educational intervention were classified under the four levels of Kirkpatrick’s hierarchy. Studies classified as Level 3 consists of simulations and observations of behaviours in activities (e.g., roleplay, standardized patients, real patients) after a learning activity such as a workshop. Although Level 3 is usually linked to students applying what they have acquired in training to job settings, our classification extends to controlled settings simulating real-life applications. The most recent search of MEDLINE Complete, CINAHL Complete and Google Scholar was carried out in October 2023.

**Table 1:** Study characteristics of the identified articles

Study Characteristics	Count (%)	References
**Publication Years**		
2004–2008	6 (31.5)	[35–40]
2009–2013	3 (15.7)	[41–43]
2014–2018	4 (21.0)	[44–47]
2019–2023	6 (31.5)	[4, 26, 29, 48–50]
**Country of Origin**		
United States	11 (57.8)	[35–41, 44–46, 48]
Germany	4 (21.0)	[4, 47, 49, 50]
Australia	1 (5.2)	[29]
Canada	1 (5.2)	[26]
New Zealand	1 (5.2)	[42]
Norway	1 (5.2)	[43]
**Types of MI**		
Adapted MI	8 (42.1)	[4, 36, 38, 42, 44, 46, 47, 49]
Group MI	7 (36.8)	[26, 29, 35, 40, 45, 48, 39]
Brief MI	4 (21.0)	[37, 41, 43, 50]
Conventional MI	0 (0.0)	0
**Medical Student Phases**		
Clinical	10 (52.6)	[4, 35, 37, 38, 41–43, 45, 46, 49]
Preclinical	7 (36.8)	[26, 29, 36, 39, 44, 48, 50]
Preclinical and Clinical	2 (10.5)	[40, 47]

**Table 2:** Detailed description of the key findings of selected articles

Author/Year	Objectives	Target Participants (Year/Phase)	Number of Participants	Delivery/Duration	Teaching methods	Assessments	Educational Outcomes	Kirkpatrick’s hierarchy (Level)
Brown et al. (2004) [36]	To determine whether a curriculum on tobacco intervention could obtain learner acceptance; improve relevant knowledge, attitudes, and self-confidence and applied to early clinical experience	(1st Year/Pre-Clinical medical students)	147 students	Face-to-face/ > 6 h	Tabacco Intervention Basic Skills (TIBS) curriculum for 1st year medical students. Learning activities include lectures, demonstrations, reading, role plays, MI quizzes, and standardized patient interview. Apply TIBS skills on actual patients.	1. Pre and Posttest Learning outcomes Questionnaires (LOQ) (18 items) to assess students’ attitudes, knowledge and self confidence in applying TIBS skills	After the intervention, 69 learners who gave pre and post intervention data showed more therapeutic attitudes and increased knowledge and self confidence in using TIBS. The posttest effect sizes were near or higher than medium strength (0.50). A follow-up two months later, 52% of 109 posttest applications had used TIBS in their clinical settings, usually for behaviours other than tobacco use. Medical students can obtain benefits from early training on encouraging behaviour change. Open-ended questions reported many participants proposed lessening workshop duration for practice and feedback and performing final skills evaluation without learner observers. 64% of students rated the TIBS curriculum as one of the top two ratings.	Level 1 and Level 2
Poirier et al. (2004) [39]	To evaluate the effectiveness of MI training on improving medical students’ knowledge of and confidence in their skill to counsel patients regarding health behavior change	(1st Year/Pre-Clinical medical students)	42 students	Face-to-Face/10 h over 5 sessions	Course on health behaviour change. Activities include small groups empathizing on learning and practicing MI techniques via brief lectures, interactive class session, learner role plays, and simulated patients.	1. Pre and Posttest to measure knowledge and confidence in performing MI 2. Assessment form to evaluate usefulness of teaching methods	The study reported medical students improved confidence in comprehending MI after the course (very confident 77%) versus pre course (2%). Significant improvement in confidence (*P* < 0.001) in all comparisons. The study showed students have significant improvement in knowledge-based questions; 31% of students answered questions correctly pre intervention, and 56% answered correctly post intervention (*P* = 0.004). This study suggests teaching MI skills to first year medical students can improve student confidence in and knowledge of providing counselling to patients on health behavioural changes. Post activity evaluation indicated teaching approaches found useful by the learners in mastering key concepts. Role-plays with simulated patients, faculty interaction with learners, and feedback were all reported by learners to be either very helpful or somewhat helpful.	Level 1 and Level 2
Mounsey et al. (2006) [38]	To determine whether utilizing standardized patients to teach this skill to 3rd year medical students would be more effective than student role plays	(3rd Year/Clinical medical students)	93 students	Face-to-Face/4 Weeks (> 10 min for each interview)	Randomized controlled trials of 3rd year medical students. Control group practiced MI. Intervention group practiced MI with standardized patients trained in MI for smoking cessation.	1. Motivational Interviewing Treatment Integrity (MITI)	Results reported were not significant between intervention and control groups in the final analysis As per MITI scores, standardized patient role-plays report similar effectiveness to student role plays when teaching basic MI skills for smoking cessation to 3rd year medical students.	Level 2 and Level 3
Martino et al. (2007) [37, 51]	To determine the effect of our curriculum on medical students’ communication skills when presented with behavioural problems during an interview, and its effect on their knowledge of and attitudes towards Brief Motivational Interviewing (BMI)	(3rd Year/Clinical medical students)	45 students	Face-to-Face/2 h	Standardized patients or instructors using teaching acronym “CHANGE” to deliver brief MI curriculum within 2 h of training to encourage students learning among medical students. CHANGE mnemonic encapsulates the basic components of BMI. In summary, facilitators trained participants to *c*heck the patient’s perspective; *h*ear the client’s views via reflective skills; *a*void MI-inconsistent behaviours such as confrontational approach and unsolicited advice; *n*ote the client’s behavioural change utilizing important and confidence ruler’s approaches; *g*ive feedback to the client only when asked; *e*nd the interview with summary of the client’s change plans and subsequent follow-up.	1. Helpful response questionnaire (HRQ) to measure their responses in different behaviour change predicaments 2. Pretest, posttest, 4 week follow up design to rate degree of which (BMI) trainers sufficiently covered the content 3. Multiple Choice Test to measure BMI knowledge	Leaners were reported to increase their usage of BMI consistent behaviours through increasing the frequency and depth of their reflections and by reducing the frequency with which they incorporated communication roadblocks and closed questions into their answer (all *P* values < 0.05). Study also reported students have increased BMI knowledge, interest to method, confidence in application of BMI in their future practice (all *P* values < 0.05). The results suggest 3rd year medical students can learn basic BMI skills and knowledge and learn positive attitudes towards the method within a relatively short duration. Future research suggests BMI curriculum evaluation studies include control groups, longer follow up evaluation, carefully coded samples of student’s skills within real patient situations and should try to link BMI proficiency to specific patient behavioural change targets. Participants suggested that their facilitators had encompassed each of the CHANGE components to a considerable degree (total mean = 6.25, SD = 1.02) with considerable to excellent skilfulness. Additionally, participants assessed the simulated patient (SP) as credibly stimulating patient behaviour change problems probable to occur in medical practice venues (mean = 7.55, SD = 1.32).	Level 1 and Level 2
White et al. (2007) [40]	To assess the effect of MI curriculum on third year students counselling skills	(1st Year and 3rd year/Preclinical and Clinical medical students)	46 students	Face-to-face/ > 3 h	MI curriculum composed of lectures, small group teaching with practice in role plays	1. MITI 2. Pre and Posttest exam related to MI 3. Student evaluation of effectiveness of MI curriculum	Study reports that MITI scores indicated that learner progressed to a proficiency level on the rate of reflections, just under the proficiency in evaluation of empathy and MI spirit and significantly below proficiency in percent of open-ended questions. The proficiency scores were meant to assess professional counsellors. However, it provided this study with preliminary results on effectiveness of the curriculum and where emphasis should be on the teaching. Evaluation of curriculum by first year MI curriculum by 112 students reported: 83% MI curriculum made them more comfortable with discussing behavioural change with patients and 93% agreed it is an important technique for physicians to possess.	Level 1 and Level 2
Bell K, Cole BA. (2008) [35]	To design and assess a formal curriculum to train medical students the principles of MI that will enhance knowledge, skills, and confidence in counselling patients for healthy behavioral change.	(3rd Year/Clinical medical students)	53 students	Face-to-face/8 h total in 4 weeks	Small groups (8–12 students). Activities include short didactics, videos, role plays and interactive exercises.	1. Pre and post assessments focusing on knowledge of MI techniques (14 item quiz), confidence in skills and satisfaction of course 2. Video Assessment of Simulated Encounter (VASE-R) 3. Questionnaire to rate the instruction of the course	Pre and posttest assessment consisted of self-reporting form measuring confidence and knowledge, and a performance assessment VASE-R tool. Knowledge (pre mean: 7.04, post mean: 11.54; *P* < 0.001, skill (pre mean: 7.04, post mean 9.47; *P* < 0.001), and confidence improved significantly (*P* < 0.001). The use of MI techniques improved in 3rd year medical students post intervention in the areas of knowledge, confidence and skills in behaviour change counselling. Future research should emphasize on patient outcomes as a measure of true effectiveness of the program. 50/53 students (93%) evaluated the teaching of the course as strongly positive (scored between a 5 or 6 on a 6-point scale, 6 being the highest quality). 49/53 students (91%) believed the course a valuable experience (scored between a 5 or 6 on a 6-point scale, 6 being the highest quality). Participant satisfaction with behaviour change program teaching increased from 3.6 to 8.1 (*P* < 0.001).	Level 1 and Level 2 And Level 3
Opheim et al. (2009) [43]	To explore the effects of brief MI for medical students	(5th Year/Clinical medical students)	113 students	Face-to-face/4 h	Workshop teaching adaptation of MI. Activities include hands-on training, brainstorming, demonstrations, sessions in groups and pairs.	1. Motivational interviewing skill code (MISC)	The study reported differences between groups were significant for five of six global MISC. Expected higher scores reported in MI group. Significant group differences in the expected direction on several behavioural measures. The intervention group had more open questions and fewer closed questions, recap, affirmed and specified patient control more, directed and had less confrontations. Four hours is insufficient to become proficient in MI. However, it is enough to have a measurable effect on medical students’ style and verbal behavior in simulated patient conditions. The participants assessed teaching in basic reflective listening as the most useful. The skills most valued by the students were: creating summaries, utilizing open questions, listening and meeting the client ‘where he stands’.	Level 1 and Level 2
Haeseler et al. (2011) [41]	To assess medical student’s MI curriculum via standardized patient case	(3rd Year/Clinical medical students)	99 students	Face-to-Face/2 h	Small group MI curriculum for 3rd year medical students which includes a didactic presentation and subsequently interactive role plays.	1. The study developed a rating scale with 8 items that links with skills taught in MI curriculum to assess MI skills.	Learners who participated in the MI curriculum were significantly reported to be more proficient than nonparticipating leaners in the performance of 2 strategic MI skills, importance, and confidence rulers (ps < 0.06). The groups did not differ in their utilization of patient-centred counselling skills or collaborative change planning. Third year medical students can learn to utilize MI skills that emphasize improving patients’ motivations for change.	Level 2 and Level 3
Lim et al. (2011) [42]	To evaluate the effectiveness of a drama training “How to act-in-role” and facilitate medical students communication in empathy	(5th Year/Clinical medical students)	149 students	Face-to-face/ > 1 h	Module with a seminar about brief intervention that provides cases of utilization of MI in doctor‒patient interaction and roleplay workshop.	1. Jefferson Scale of Physician Empathy (JSPE) 2. Objective Structured Clinical Examination (OSCE) 3. Behavior Change Counselling Index (BECCI)	The baseline of both groups did not differ in empathy scores measured by the JPSE. The intervention group showed significantly higher empathy scores after the intervention. The intervention group were also rated significantly higher in tutor ratings for their MI via BECCI and overall OSCE performances. The intervention was successful in improving self-reported empathy scores among medical students and their competence in consultation techniques. Future research suggested is via actor facilitated teaching innovation.	Level 2
Kaltman et al. (2015) [46]	To demonstrate the feasibility and effectiveness of an enhanced MI curriculum within a required 4-week Family Medicine clerkship To determine the feasibility of giving training to learners in the content of only minimal face to face time	3rd Year/Clinical medical students)	17 students	Blended Learning/4 Week Clerkship (> 1 and half hours for face-to-face content) Online component is up to discretion of student	Online learning community for enhanced MI for 3rd year medical students Website materials include MI, problematic health behaviours, repository of exemplar videos and students’ videos with feedback and discussion board.	1. Demographic Questionnaire 2. Questionnaire assessing confidence in MI Skills adapted from Poirier et al. (2004) 3. OSCE with Standardized patient to assess MI skills using an Adapted MITI.	Learners in the enhanced training arm have significantly higher scores in MI style in OSCE than training as usual students A significant increase in self-efficacy from pretest to posttest in the overall sample was observed but between group differences were insignificant. Results of this study and the student feedback suggest that future research include patient practice sessions and individualized feedback in developing motivational curricula. Other suggestions for research, for future physicians to achieve proficiency in MI focused instruction and sufficient chance to practice is needed. Student feedback was especially positive regarding video recorded practice sessions with patients and individualized feedback.	Level 1 and Level 2
Brogan et al. (2016) [44]	To aim is to provide an advanced form of patient centred communication via an MI curriculum.	(1st Year/Pre-Clinical medical students)	120 students	Face-to-face/12 h	MI Workshop via small group activities (SGA), and large group lecture (LGA). Sessions include reading articles, watching videos, group role pay, MI introductory demo.	1. MITI 2. Questionnaire feedback on MI on course objectives, content, self-rated improvement skills and relevance to MI to medical education.	Significant improvement in preliminary pretest and posttest training scores from medical students and display significant improvement to expression of empathy and ratio of reflections to questions. Implementation of the MI interviewing curriculum has given learners the chance to practice evidence-based communication that encourages motivation for health behavior transition in patients, with emphasis on chronic disease management. Positive feedback on student evaluation forms in areas of relevance to training and self-rated skills improvement.	Level 1 and Level 2
Gecht-silver et al. (2016) [45]	To assess changes in 3rd year medical students’ knowledge, skills and attitudes from 4 h of MI training	(3rd Year/Clinical medical students)	53 students	Face-to-face/4 h	MI workshop. Activities included are discussion, lecture, practical session (MI skills and OARS – open ended questions, affirmation, reflective listening, and summary) and homework.	1. VASE-R 2. MI knowledge questionnaire 3. Attitude Questionnaire	Data were analysed using t test analysis and qualitative thematic analysis. Participants showed significant improvement in confidence, knowledge, and skills. This study suggests that 4 h of training can give positive changes to medical students and supports its use in medical education programs. Future studies can include assessment of curriculum enhancements with a more rigorous research design and development of additional training chances. Students’ qualitative feedback showed increased understanding of MI and the want and confidence to utilize new skills.	Level 1 and Level 2
Purkabiri et al. (2016) [47]	To teach learners on working effectively with patients in the region of smoking cessation counselling through an efficient 4-h comprehensive course	(1st Year to 6th Year/Preclinical and Clinical medical students)	88 students	Face-to-face/4 h	Medical students from 1st year to 6th year medical were taught by a doctoral student in five identical 4 h courses Materials include: 45-min theoretical introduction, role playing, a pre and posttest assessment for knowledge, skill, and attitude.	1.Pre and Post 4-week training questionnaires to assess knowledge, skills, and attitude 2. Blinded analysis of pre and post course videos of 5-min standardized patient situation to assess knowledge, skills, and attitudes	In terms of Knowledge assessed: Before the intervention 10.6 (mean, SD:2.7) question of 29 were answered with correct answer and increased to 19.2 (3.6) after the course (*p* < 0.0005). The Cohen’s d effect size was 2.7. Major features of the medical students’ counselling skills improved with Cohen’s d effect size of 1.3. There are significant and highly relevant attitude changes shown in increased motivation to counselling smokers. The incorporation of a 4-h smoking cessation workshop into the curricula was reported to be highly effective in enhancing student’s knowledge, skills and attitudes about smoking counselling, alongside giving them other clinical competencies. Most students reported on a visual analogue scale that they strongly agreed (strongly agree = 0–19 pts; mean = 174; SD = 3.8) with the statement “whether they think that it would be possible to counsel smokers on the basis of the knowledge and skills in the course”.	Level 1 and Level 2 And Level 3
D’Urzo et al. (2020) [26]	To assess the implementation and impact of an MI workshop on 2nd year medical students MI knowledge and Theory of Planned Behavior (TPB) social recognitions for	(2nd Year/Preclinical medical students)	27 students	Face-to-face/3.5 h	MI Workshop. Activities include lecture, experiential learning, seminar, workshop summary and debrief	1.Demographics Questionnaire 2. Questionnaire assessing satisfaction in learning opportunities adapted from the Course Experience Questionnaire (CEQ)	Learners showed a significant increase in MI knowledge from pre and post workshop (*p* = 0.001). Not insignificant, small to moderate effect sizes in change in social cognitions were reported from pre to post workshop. Medical students view MI in high regard, as shown by the relatively high cognitions reported in the baseline reports preworkshop. Future research should focus on future workshops that focus on allocating more time for skill acquisition for proficiency in clinical use. The students reported the workshops of high quality (all average feedback scores were than > 3.9/5). The overall average feedback rating for each tutor was within a range of 4.3 to 4.9/5.	Level 1 and Level 2
Keifenheim et al. (2019) [49]	To evaluate the medical student’s interest in MI, objective need for training, and learner satisfaction with effectiveness of implemented course	(6th Semester/Clinical medical students)	114 students	Blended Learning/ > 2 h and 45 min	Utilizing 6 MI classroom-based lessons, mandatory video materials between sessions containing MI spirit, interviewing skills, agenda mapping and explore goal and values. Online content for discussion. Classroom sessions include role plays and consultation with simulated patient and performing basic MI skills.	1. Questionnaire on MI experience and evaluation of the new course module 2. Multiple-choice test to test knowledge on MI 3. The Calgary-Cambridge Observation Guide (C-CG) 4. MITI	Learners were highly interested in learning MI. Preintervention showed good communication and moderate MI skills. The effectiveness of the course was assessed via significant improvement in subjective, objective knowledge and skills Further research should deliberate on giving students special training in MI to assist students in counselling patients towards behavioural change. Participants reported being very interested in studying a behaviour-change counselling approach and found it useful. The participants rated the recently created course module as good (2) on a rating scale between 1 to 6. Satisfaction with the course was high.	Level 1 and Level 2
Jacob et al. (2021) [48]	To determine the effectiveness of a case-based curriculum on MI for preclinical medical students	(1st Year/Pre-Clinical medical students)	68 students	Face-to-face/8 h	2 h didactic sessions, with 3-h case-based sessions that include role plays	1. Pre and Post training questionnaires to assess knowledge, attitudes, and self-efficacy 2. Questionnaire to assess satisfaction of program	Study reports significant improvement of MI, attitudes regarding MI in health care venues, self-efficacy, and conversing with patients about behaviour change. The case-based curriculum on MI was effective in helping preclinical students to learn the knowledge and skills required to deliver MI in a wide range of cases. Students were also highly satisfied with the MI training (M of 4.4, SD = 0.6/0.5)	Level 1 and Level 2
Plass et al. (2022) [50]	To assess the effectiveness of a brief virtual role, play MI training program on MI knowledge and skills medical students	(1st Year/Pre-Clinical medical students)	339 students	Online/1 h	Four 10 to 15 min MI-games based on training conversations in the Kognito conversation Platform were presented to learners using a single group- Interrupted Time Series Design. Activities included, learners assumed the role of a healthcare entities and carried out role plays with virtual patients who simulate human behaviour.	1. The study developed a pretest and retrospective pretest on both MI knowledge and skills 2. Self-report questionnaire of the program	Utilizing a pretest and then-test (retrospective test) to evaluate response shift in assessing the educational intervention. The one-hour MI virtual training showed effectiveness in two areas: learners gained knowledge and skills, improved awareness of the existing intrinsic knowledge and skill they can use for future patients in a patient centred approach. A brief one-hour MI training simulation is effective even early on in medical education. Future research should focus on whether the effect found consolidates over time, and what way raising awareness of internal MI skills contributes to future patient centred communication skills. The overall rating of the course in terms of mean score is 2.8 (SD = 0.73; *N* = 325). Most of the participants (69%) graded ‘good’ (3) or excellent (4). A minority of participants (4%) rated ‘poor’.	Level 1 and Level 2
Edwards et al. (2022) [29]	To assess teaching brief MI to preclinical medical students	(Preclinical medical students)	46 students	Face-to-face/12 h over 8 weeks	Educational intervention based on the Learn, See, Practice, Prove, Do, Maintain Pedagogical Framework. Activities include lectures, role play, small group simulated patient encounters with scaffolding strategies	1. Motivational interviewing confidence scale (MICS) 2. Motivational Interviewing Knowledge and Attitudes Test (MIKAT) 3. Multiple Choice Knowledge Test to measure knowledge 4. BECCI	Medical students who receive brief MI training, gained knowledge and confidence from baseline and post training and gains remained at 3 months. Brief MI skills were reported to improve across simulation sessions. Preclinical students can obtain knowledge, confidence, and skills in brief MI after attending a brief intervention and improvements are maintainable.	Level 2
Erschens et al. (2023) [4]	To examine the success of MI via a blended learning format for medical students in between their 6th and 9th semesters before the Covid-19 pandemic	(6th to 8th Semester Students/Clinical medical students)	49 students	Blended Learning/5 h 45 min over 6 sessions	The course is divided into 6 sessions. The first session is a seminar on “Psychosomatic Medicine and Psychotherapy”. Online teaching and demonstration videos were also given to students. The teaching videos showed students exemplary conversations of doctors applying MI to simulated patients. Other sessions were incorporated into existing psychiatry postings.	1.Knowledge Test to evaluate each curriculum unit of MI 2. Evaluation of subjective theoretical knowledge and practical skills in MI 3. Evaluation of curriculum satisfaction by learners	The data from the control (*n* = 14) and intervention group (*n* = 35) were analysed with a participation rate of 65.7%. Overall interest in studying MI was high, M = 2.92 (SD = 1.00). This suggests there is enhanced knowledge over time in the intervention group. The study reports the effectiveness of an MI curriculum for medical students. The incorporation of MI into the curricula in medical school is a potential circular addition to enhance doctor‒patient communication. 62.8% of medical students said the curriculum was significant to their future profession. High level of satisfaction with practical relevance was reported via free- form text responses.	Level 1 and Level 2

## Results

**Fig. 1:**
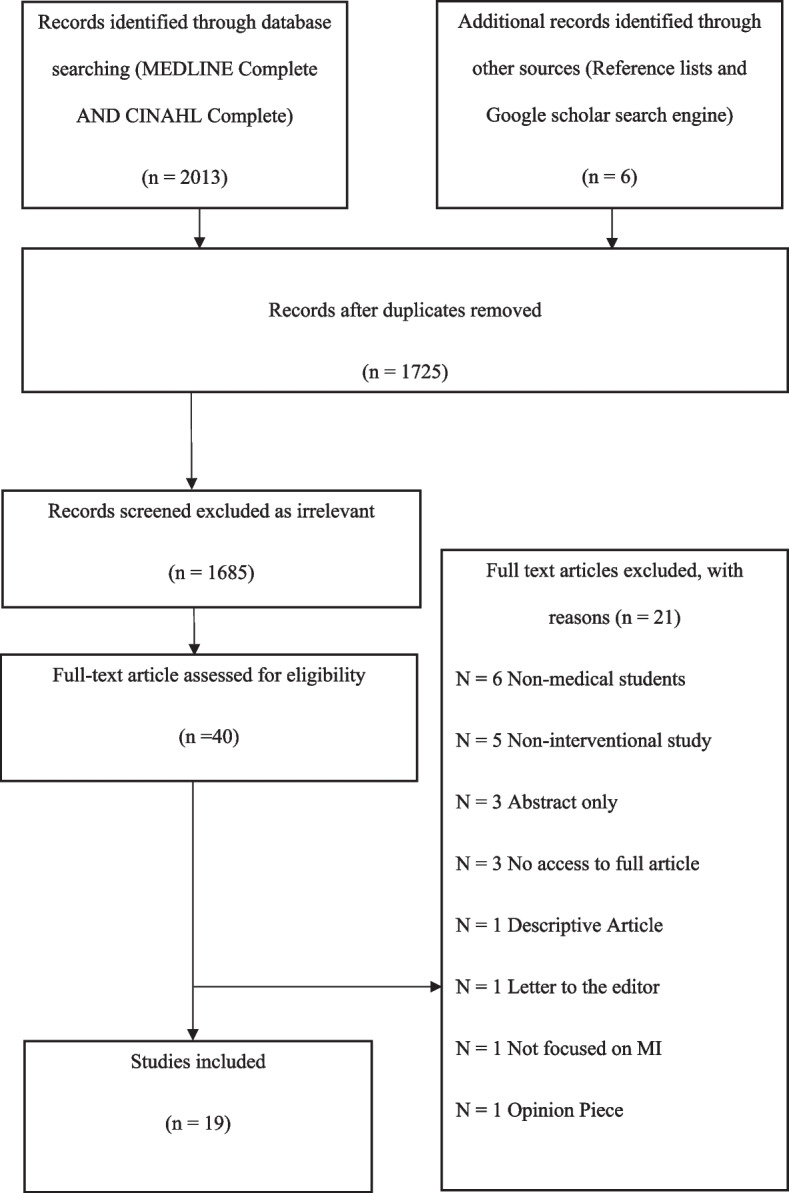
Prism flow diagram

From an initial pool of 2,019 articles, after removing duplicates and screening for relevance, 19 articles were included in this review. The detailed selection process is illustrated in the PRISMA flow diagram in Fig. 1.

### Characteristics of the identified articles

The study characteristics, country of origin, and phase of study are presented in Table 1. The detailed descriptions of the key findings of these articles (i.e., author, year, objectives, participants, delivery, duration, teaching methods, assessments, and educational outcomes based on Kirkpatrick’s hierarchy) are provided in Table 2. Most of the studies were published between 2004–2008 and 2019–2023, with each period accounting for 31.5% of the total articles. The majority of MI studies originated from the US (57.8%).

### Types and characteristics of MI

With respect to the first research objective, none of the 19 studies in this scoping review conducted conventional MI. Rather, most studies in this scoping review used adapted MI (*n*=8; 42.1%) [4, 36, 38, 42, 44, 46, 47, 49], followed by group MI (*n*=7; 36.8%) [26, 29, 35, 40, 45, 48, 39] and brief MI (*n*=4; 21%) [37, 41, 43, 50].

Adapted motivational interviewing was utilized in 8 studies. This approach includes any adaptations utilized to adjust MI culturally to the situation or facilitated by technology via different types of content and technologies (e.g., computers, smartphones, applications, videos and audio). Additionally, it also includes adaptations made to structured curricula, such as using role plays via standardized patients or real patient interactions to facilitate the learning of MI. Adapted MI was reported in 8 studies. Specifically, 5 studies [36, 38, 42, 44, 47] adapted their curricula to teach MI via role playing standardized patients or real patients. Additionally, 3 studies [4, 46, 49] utilized technological adaptations and blended learning (face-to-face and online) to teach motivational interviewing.

In group MI, this approach consists of MI that is adapted for group format and is MI consistent (e.g., applying MI principles, spirit and techniques in its delivery). Group MI was carried out in 7 studies. Two studies [26, 45] used training workshops to teach and practice MI in smaller groups. The remaining 5 studies [29, 35, 39, 40, 48] used a small group format to teach MI skills consisting of lectures, roleplay, a case-based curriculum and demonstrations.

Brief MI provides brief consultations centred on typically shorter number sessions (e.g., 1--2 sessions) than conventional MI (e.g., 3--4 sessions or more). A brief MI was conducted in 4 studies. Two studies [41, 51] delivered a single session of MI training within two hours. Another study [50] conducted four (10–15 minute) sessions teaching MI, with a total of less than 1 hour of training. Opheim et al. [43] conducted a four-hour workshop on MI, which is a relatively brief training intervention.

More than half of the studies focused on clinical medical students (*n*=10; 52.6%) [4, 35, 37, 38, 41–43, 45, 46, 49], and the least studied was the combination of preclinical and clinical students (*n*=2; 10.5%) [40, 47]. There was a diverse number of participants, ranging from 17 to 339 students. The median number of participants in these studies was 93. The most common delivery mode identified was face-to-face learning (*n*=15; 78.9%) [26, 29, 35, 36, 38–45, 47, 48, 51], followed by blended learning (*n*=3; 15.7%) [4, 46, 49], and the least common delivery mode was online learning (*n*=1, 5.2%) [50]. The duration of intervention for brief MI (*n*=4; 21.0%) [37, 41, 43, 50] ranged from 10 minutes to 2 hours per session. The duration of adapted MI (*n*=8; 42.1%) [4, 36, 38, 42, 44, 46, 47, 49] and group MI (*n*=7; 36.8%) [26, 29, 35, 40, 39, 45, 48] ranged from 3 hours to 12 hours. The teaching methods include workshops, lectures, videos, role plays, demonstrations, interviews, interactive exercises, small and large group activities, simulated patients, and online forums.

### Classifying educational outcomes based on Kirkpatrick’s hierarchy

With respect to the second research objective (i.e., classifying educational outcomes on the basis of Kirkpatrick’s hierarchy [32]), all 19 studies [4, 26, 29, 35, 36, 38–51] were categorized at Kirkpatrick’s Level 2 (knowledge/skills/attitudes). This is followed by 16 out of 19 studies [4, 26, 29, 35, 36, 39, 40, 43–51] categorized at Kirkpatrick’s Level 1. Only 4 out of 19 studies [35, 38, 41, 47] are categorized at Kirkpatrick’s Level 3 (Behaviour). One of the studies [38] compared the effectiveness of standardized patients versus role plays from colleagues and reported that both were equally effective for teaching basic MI skills among medical students. The students were evaluated in a simulated environment and demonstrated their MI skills in terms of student roleplay or standardized patients. The study reported that standardized patient role play is as effective as student role play in teaching basic MI skills. The sessions focused on demonstrating skills in a simulated setting, suggesting that the student’s behaviour (i.e., adherence to MI skills) was evaluated and improved via the educational intervention. In another study, Bell et al. [35] investigated the use of a curriculum to teach medical students the principles of MI to increase their knowledge, skills and confidence in counselling patients with the aim of health behaviour change. The research indicated that video-recorded interactions between students and patients enabled students to effectively apply MI skills to real-life patients. None of the studies included reported outcomes at Level 4 (results).

### Key elements of the reported FRAMES model and assessment methods used

**Fig. 2:**
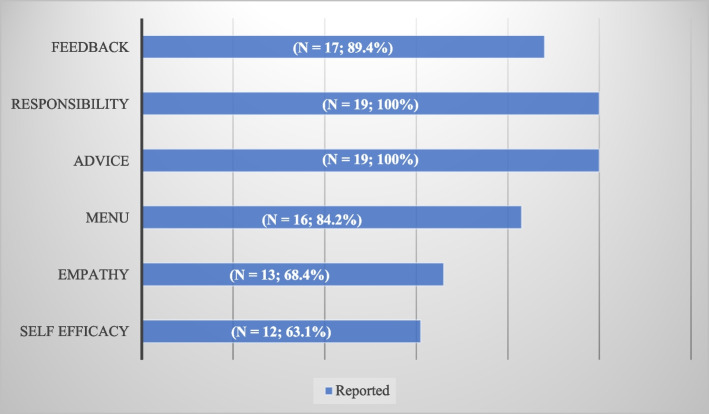
Important elements of MI interventions (*n* = 19) identified as “reported” via the FRAMES model

With respect to the third research objective, all 6 elements in the FRAMES model were covered in 9 out of 19 studies [4, 29, 35, 36, 39, 40, 44, 45, 51], 5 elements were identified in another 4 studies [26, 41, 48, 49], and 4 elements were identified in 4 studies [42, 43, 46, 47]. The most reported element in all 19 studies was responsibility and advice (*n*=19; 100%), and the least reported element was self-efficacy in only 12 studies (*n*=12; 63.1%) [4, 29, 35, 36, 39–41, 44–46, 48, 51]. Figure 2 shows additional details on the important elements present in the MI interventions.

The primary assessment method used across the studies was the use of pre- and posttest surveys, which are used to measure knowledge (*n*=10, 52.6%), skills (*n*=5, 26.3%) and attitudes (*n*=3, 15.8%) pertaining to MI. Moreover, the specific instruments employed for focused assessments were (1) MITI to measure fidelity of MI in 5 out of 19 studies (*n*=5, 26.3%), (2) Video Assessment of Simulated Encounters (VASE-R) to measure MI skills in 2 out of 19 (*n*=2, 10.5%) (3) Behaviour Change Counselling Index (BECCI) to measure practitioner’s skill and competence in delivering effective MI in 2 studies out of 19 (*n*=2, 10.5%), (4) Objective Structured Clinical Examination (OSCE) to measure clinical competence in 2 studies out of 19 (*n*=2, 10.5%), (5) Motivational Interviewing Knowledge and Attitudes Test (MIKAT) to measure the practitioner’s knowledge and attitude pertaining to MI in 1 study out of 19 (*n*=1, 5.2%), (6) Motivational interviewing skill code (MISC) to measure adherence to MI in 1 study out of 19 (*n*=1, 5.2%), (7) the Calgary-Cambridge Observation Guide (C-CG) to measure communication skills between practitioners and patients was used in 1 study out of 19 (*n*=1, 5.2%), (8) Motivational interviewing confidence scale (MICS) to measure confidence in health behaviour change dialogues in 1 study out of 19 (*n*=1, 5.2%) and (8) the Jefferson Scale of Physician Empathy (JSPE) to measure empathy in patient care among health practitioners in 1 study out of 19 (*n*=1, 5.2%).

## Discussion

Our scoping review sheds light on the current trends and key findings to determine the types of MI education programs in medical schools, the delivery modalities and teaching methods used, classify educational outcomes on based on Kirkpatrick’s hierarchy [32] and determine the key elements of MI education covered via the FRAMES model. First, there appears to be a bimodal distribution of most articles published between the two time periods of 2004--2008 and 2019--2023. Second, all the studies included in this review did not use conventional MI but instead utilized a variety of MI adaptation techniques. Third, most studies used face-to-face training in MI, whereas only one study used online delivery. Fourth, most studies have used a variety of interactive experiences to teach MI. Next, all studies reported outcomes at Kirkpatrick’s Level 2, but only 4 studies reported outcomes at Kirkpatrick’s Level 3. Finally, the most covered elements of MI training in these studies were responsibility and advice (*n* = 19; 100%), and the least covered element in MI training was self-efficacy (*n* = 12; 63.1%) [4, 29, 35, 36, 39–41, 44–46, 48, 51]. This review expands on the evidence of MI interventions among medical schools. The results of our findings generally suggest that MI can be effectively taught in medical schools. Furthermore, we have provided several recommendations for further research to improve the implementation of MI in medical schools.

There appears to be a bimodal distribution of published articles between the two time periods, i.e., between 2004 and 2008 and between 2019 and 2023. A decline in the number of articles published was observed between 2009 and 2019. This decline could be due to the shift in the applications of MI beyond treating addictive behaviours to include a broad range of other behavioural conditions [52], such as its expanded applications in school education [53–55], lifestyle coaching [56–58], probation and parole [59, 60] and digital health care and telemedicine [61, 62]. From 2019 onwards, however, there was an increasing trend in the number of published articles on MI training for medical students. This could be attributed to the MI Network of Trainers (MINT) making it mandatory to attend MI training during the COVID-19 pandemic to provide virtual training in 2020 and 2021 [52], which has facilitated remote participation.

### Types of MI education programs in medical schools

All the studies included in this review did not use conventional MI but utilized a variety of MI adaptation techniques. Most studies [4, 36, 38, 42, 44, 46, 47, 49] have used adapted MI to conduct their MI training, possibly because of the need to tailor MI programs to fit medical school curricula. Medical students have been linked to extensive academic responsibilities and clinical rotations [63], contributing to this adaptation of MI. In fact, the lack of harmonization of training methods among medical schools has led to challenges in understanding the optimal approach to teach MI among medical students [31]. Furthermore, there is no consensus on the standard dose of training in MI that is adequate or mandatory for learners to acquire sufficient skilfulness in the practice of MI [9]. Moreover, medical schools have time constraints and limited MI teaching opportunities because of their hectic medical curriculum schedules [41]. This may lead to a variety of adaptations of MI, as noted in this review. Future research can focus on addressing the lack of harmonization in MI training methods and emphasize building and employing standardized MI training with adequate dosing across medical schools.

### Delivery modalities and teaching methods used

In the present review, the delivery modalities used to train medical students in MI varied across the studies. Most studies [26, 29, 35, 36, 38–45, 47, 48, 51] have focused on delivering face-to-face training on MI to clinical medical students. This aligns with the current literature, which suggests that MI is a complex communication skill [57] and is reported to be taught more effectively in face-to-face sessions [64]. In this review, only one study [50] used a fully online approach to teach MI to medical students. A systemic study suggested that for an online MI intervention to be effective, it requires significant emphasis on fidelity and training procedures [65]. In a recent comparative study, Schaper et al. [66] reported similar effects of training MI among general practitioners in both online and face-to-face training in MI skills and spirit. Future studies could focus on the implementation of online versus face-to-face training for medical students with an emphasis on fidelity and training procedures for MI.

A large proportion of the studies in this review report the use of a variety of teaching approaches (e.g., workshops, role-play, standardized patients, and small and large group sessions) to teach MI. This aligns with Kolb’s experiential learning cycle [67], where the process of learning occurs when knowledge is formed via the transformation of experience. This model is guided by four phases of the learning process: concrete experience (having an experience), reflective observation (reflecting on an experience), abstract conceptualization (learning from the experience), and active experimentation (experimenting what you have learned). Medical students who are given the opportunity to engage in Kolb’s learning cycle [67] via interactive activities, reflection and simulated or real-life settings are likely to develop good MI skills. Future research should underpin educational theories into MI training by implementing structured reflective exercises in MI education.

### Educational outcomes based on Kirkpatrick’s hierarchy

Our review shows that all studies reported outcomes at Kirkpatrick’s Level 2, suggesting that medical students have acquired the intended knowledge, skills, and attitudes. There are only 4 studies that reported outcomes at Kirkpatrick’s Level 3, which evaluates the degree to which the students apply their learning to simulated or real-world settings. The first 3 studies [38, 41, 47] showed their improvement in behaviour by showing their learned skills in realistic settings, which included observing students’ behaviour in standardized patients or real patients. The last study [35] revealed improvements in the MI skills of real patients in diverse settings, such as traditional health behaviour interventions, such as alcohol, tobacco and weight loss interventions. Future studies should include longitudinal evaluations of the effectiveness of MI skills.

### Key elements of MI education covered via the FRAMES model

According to the FRAMES model [68], all included studies reported the elements of responsibility and advice (*n*=19; 100%) in the training of MI. The element responsibility is the shared responsibility of the learner’s growth by the learner and teacher. This could be attributed to the move towards competency-based medical education, which emphasizes shared responsibility among students while incorporating student-centric learning techniques and formative assessment as a vital element of the learning process [69]. In other words, the high reporting of ‘responsibility’ and ‘advice’ suggest that the present MI training significantly emphasizes medical students taking ownership of their learning and decision-making processes (‘responsibility’). Moreover, from a patient education perspective, empowering patients to take ownership of their health [70] and effectively guiding patients toward positive behavioural changes through good advice in a nonconfrontational approach is a basic tenet of MI (‘advice’).

The least reported element found in training for MI in our included studies [4, 29, 35, 36, 39–41, 44–46, 48, 51] was self-efficacy. This may be due to MI training focusing less on self-efficacy and instead emphasizing other elements, such as empathy, open-ended questioning and reflective listening. An educational theory that is linked to the element of self-efficacy is social cognitive theory. Social cognitive theory can be defined as a person’s belief in their ability to determine the behaviours required to reach their desired goals and their perceptions of their ability and skills to manage their environment [71, 72]. Continued research into integrating social cognitive theory into MI training could assist practitioners in comprehending the role and importance of self-efficacy in behaviour change and reflective practice. The lower reporting of ‘self-efficacy’ might also indicate a potential gap in MI training. Self-efficacy is essential because it relates to the practitioner’s confidence in their ability to effectively implement MI techniques and facilitate behaviour change in patients. Addressing this gap in future research could lead to more competent and confident practitioners who are better equipped to address challenging patient interactions and support positive health outcomes. Future studies can also utilize FRAMES to guide research design and interventions and investigate which aspects of FRAMES in the training of MI are most effective within the limited time frame of medical curricula.

## Limitations

This scoping review is subject to several limitations. We included only English-language studies in which medical students were the target participants. We did not include articles that are categorized as grey literature or other forms of nonpeer review articles, which might have resulted in biased outcomes. Most of the studies focused on evaluating learner knowledge and skills in MI, which might have limited the practical applications of MI to real patients. The first author conducted the search and screening of the articles. This may lead to selection bias and reduce the reliability of the study selection process. The protocol for this review was developed before the search was initiated but was not registered or published online, which increases the risk of selective reporting. The database search was limited to MEDLINE Complete and CINAHL Complete, which were accessed via EBSCOhost and the search engine Google Scholar. Although a comprehensive search was conducted, other databases that were relevant to the review, such as the PsycINFO and ERIC databases, were not included, potentially resulting in missing relevant articles. Kirkpatrick’s hierarchy was utilized to assess educational outcomes in this review. This approach may neglect other core aspects of educational interventions. Furthermore, although we have extensively searched various countries, most of the studies reported are from the USA (*n*=11; 57.8%) or Germany (*n*=4; 21.0%). A lack of diversity among studies in other regions may lead to biased outcomes.

## Conclusion

Based on our review, the findings suggest that motivational interviewing can be taught effectively in medical schools via adaptations of MI and a variety of teaching approaches. However, there is a need for further research investigating standardized MI training across medical schools, the adequate dose for training in MI and the implementation of reflective practices that are supported by educational learning theories. Furthermore, longitudinal studies can assess the effectiveness of MI. Future studies may benefit from exploring and better understanding the relationship between MI and self-efficacy in their MI interventions. The FRAMES model can be used to guide research and explore which aspects of FRAMES are optimally delivered within the limited time frame of medical curricula.

## Data Availability

All data generated or analysed during this study are included in this published article.

## Abbreviations

ADHD: Attention-deficit/hyperactivity disorder
BECCI: Behaviour Change Counselling Index
BMI: Brief motivational interviewing
CEQ: Course Experience Questionnaire
C-CG: Calgary-Cambridge Observation Guide
CINAHL: Cumulative Index of Nursing and Allied Health Literature
FRAMES: Feedback, Responsibility, Advice, Menu of Options, Empathy, Self-Efficacy
HIV: Human immunodeficiency virus
HRQ: Helpful response questionnaire
JPSE: Jefferson Scale of Physician Empathy
LGA: Large Group Activities
LOQ: Learning Outcomes Questionnaire
MEDLINE: Medical Literature Analysis and Retrieval System Online
MI: Motivational interviewing
MICS: Motivational Interviewing Confidence Scale
MIKAT: Motivational Interviewing Knowledge and Attitudes Test
MINT: Motivational interviewing network of trainers
MISC: Motivational interviewing skill code
MITI: Motivational interviewing treatment integrity
MMI: Mechanisms of Motivational Interview
OARS: O = open-ended questions, A = affirmations, R = reflections, and S = summaries to promote active listening
OSCE: Objective Structured Clinical Examination
PRISMA-ScR: Preferred Reporting Items for Systematic reviews and Meta-analysis extension for Scoping Reviews guidelines
SGA: Small Group Activities
SP: Simulated Patient
TIBS: Tabacco Intervention Basic Skills
TPB: Theory of Planned Behaviour
VASE-R: Video Assessment of the Simulated Encounter
